# The Effect of Exercise Compliance on Risk Reduction for Hamstring Strain Injury: A Systematic Review and Meta-Analyses

**DOI:** 10.3390/ijerph182111260

**Published:** 2021-10-27

**Authors:** Nicholas Joel Ripley, Matthew Cuthbert, Steven Ross, Paul Comfort, John James McMahon

**Affiliations:** 1 Human Performance Laboratory, University of Salford, Salford M5 4BR, UK; M.Cuthbert@edu.salford.ac.uk (M.C.); S.Ross6@edu.salford.ac.uk (S.R.); P.Comfort@salford.ac.uk (P.C.); j.j.mcmahon@salford.ac.uk (J.J.M.); 2 The FA Group, St George’s Park, Burton-upon-Trent, Staffordshire DE13 9RN, UK; 3 Department of Sport and Physical Activity, Edge Hill University, Ormskirk L39 4QP, UK; 4 School of Medical and Health Sciences, Edith Cowan University, Joondalup, WA 6027, Australia; 5 Institute for Sport, Physical Activity and Leisure, Carnegie School of Sport, Leeds Beckett, Leeds LS1 3HE, UK

**Keywords:** hamstring strain injury, risk reduction, compliance, consistency, modality, nordic hamstring exercise

## Abstract

Eccentric strength training can reduce the risk of hamstring strain injury (HSI) occurrence; however, its implementation can be impacted by athlete compliance and prescription. The aim of this review was to investigate the effects of intervention compliance, consistency and modality, on the prevention of HSIs among athletes. A literature search was conducted. 868 studies were identified prior to the application of the exclusion criteria which resulted in 13 studies identified. Random effects models were used to produce log odds ratios and 95% confidence intervals. Very high (>75.1%), moderate-high (50.1–75%), low-moderate (25.1–50%) and very low (<25%) and <1-, 1.01–3.00-, >3.01-weeks/session were used as thresholds of compliance and consistency, respectively. Modality was also observed. A positive effect on HSI incidence -0.61 (−1.05 to −0.17), favoring the intervention treatments (Z = −2.70, *p* = 0.007). There were non-significant, large differences between compliance (*p* = 0.203, Z = −1.272) and consistency (*p* = 0.137, Z = −1.488), with increased compliance and consistency showing greater effectiveness. A significant difference between intervention modalities was observed (*p* < 0.001, Z = −4.136), with eccentric interventions being superiorly effective. Compliance of >50.1% and consistent application with <3 weeks/session having positive effects on HSI incidence. Training interventions that can achieve high levels of compliance, and can be consistently performed, should be the objective of future practice.

## 1. Introduction

The alarming incidence and cost (both financial, up to €500,000/month in elite soccer and time-loss, 15–20 days missed) of sustaining a hamstring strain injury (HSI) demonstrate the need to intervene [1,2,3], with appropriately designed training interventions that have the ability to reduce the occurrence of HSIs. Researchers have previously identified that the implementation of strength training, including eccentric exercise has the ability to reduce the risk of future HSI occurrence [4,5,6,7]. However, Bourne et al. [8] highlighted that the resultant risk reducing benefits only occur when an adequate intervention compliance is achieved, although the effect of compliance levels on desired outcomes is yet to be quantified. A key issue within elite sport is that evidence based HSI prevention exercise; namely, the Nordic hamstring exercise (NHE) is not being adopted by many elite soccer and cricket teams [1,9,10]. The primary complaint by players and coaches of both soccer and cricket is delayed onset muscle soreness (DOMS), as a result of the eccentric nature of the NHE [1,9,10,11,12]. There are, however, methods to offset DOMS via a progressive introduction of the NHE, which could be achieved by using low volumes and lower intensities at any given knee angle by performing incline NHE variations [13,14], as the magnitude of the repeated bout effect appears to be similar between high and low volumes of eccentric exercise [15]. Hence, a training intervention that facilitates a wider scale adoption, with improved compliance rates, while concurrently reducing the incidence of HSIs requires exploration.

A recent review recalibrated the previously identified risk factors associated with HSI incidence, providing a similar conclusion by identifying that measures of strength and muscle architecture are key modifiable risk factors for HSI incidence [16]. The two most common practices to reduce HSI that have been incorporated into sport involve the implementation of an eccentric hamstring strengthening exercise on its own [6], or as part of specific warm up protocols, such as the FIFA 11 and FIFA 11+ for soccer [17,18]. These approaches have become common partly due to the positive adaptations that are known to occur from the implementation of the NHE [19,20,21,22,23,24,25,26] and the relative ease of its implementation, due to it requiring very limited time and equipment. The evidence highlights that there is a need for high compliance rates, where an intervention can maintain participant involvement throughout a training period, as there is a rapid detraining effect from cessation of the NHE, with decreases in hamstring muscle architecture and strength reported within as little as two weeks of cessation [25,26,27,28]. Therefore, regular or consistent performance of the NHE, or similar exercises, with high compliance is essential to maintain positive adaptations.

The utilization of the NHE within training or the FIFA 11 and FIFA 11+ has been extensively examined in meta-analyses [18,29,30,31]. In fact, systematic reviews continue to be published despite consistent findings, with very limited change in the studies observed between them [18,29,30,31]. The consistent findings of these meta-analyses demonstrate that eccentric resistance training and the FIFA 11+ have the potential to decrease the occurrence of HSI in athletic populations [18,29,31] by up to 50% [30]. The notion that HSIs can be reduced by up to 50% has recently been questioned in the literature; Impellizzeri, McCall and van Smeden [32] identified several methodological inaccuracies preventing replication of this result, suggesting that the NHE can only be conditionally recommended [32]. Despite the inconclusive findings, interventions remain effective at reducing the occurrence of HSI in athletic populations [18,29,31]. However, the adoption and implementation of such interventions is continually reported to be “adequate” at best [8], with compliance being considered a key component for an effective eccentric resistance training intervention aimed at reducing HSI incidence [29]. Similarly, for the FIFA 11+, <15% of teams completed the recommended volume; as such, this compromises the risk-reducing effectiveness of the FIFA 11+, in addition to the resultant risk ratios reported within the meta-analysis [18]. Goode et al. [29] further identified that with increased compliance there was a 65% decrease in the risk of HSI occurrence; however, no systematic review to date has quantified what an adequate level of compliance is for an intervention to be deemed effective. Grouping of studies in accordance with compliance to any injury risk reducing intervention protocol has been used previously; van Reijen et al. [33] differentiated studies by <24.7%, 24.8–48.1% and >48.2%. However, given the huge importance of reducing HSI in athletic populations [2,3], a higher compliance should be aimed for in HSI prevention interventions as even the “high level” of 48% compliance could lead to a prolonged period without an intervention stimulus, potentially reducing muscle architectural and eccentric strength adaptations [25,26,27,28]. Therefore, novel compliance thresholds require identification for practitioners, which could be used as a guide to the implementation of appropriate and effective training practices that could reduce the risk of HSI incidence.

To date, quantifying the effect of intervention compliance on HSI risk has never been performed, despite commentary that achieving a high level of intervention compliance is crucial in reducing injury risk [8]. Therefore, the purpose of this systematic review and meta-analysis is to identify the randomized control trials (RCT) that have examined the effects of HSI prevention programmes’ (that hypothesised to increase bicep femoris fascicle length and/or the strength of the hamstrings or associated structures) compliance, consistency and modality, on the prevention of HSIs among athletes.

## 2. Materials and Methods

The Preferred Reporting Items for Systematic Reviews and Meta-Analyses (PRISMA) guidelines were used in the development of the present systematic review and meta-analysis. The 27-item checklist identified within the PRISMA statement is designed to be used as a basis for reporting randomized trials [34]. A review protocol was not registered for this review.

### 2.1. Search Strategy

A systematic, computerized search of the literature in PubMed, SPORTDiscus, MEDLINE, Scopus and Web of science was conducted, with controlled vocabulary and key words related to hamstring injury prevention programmes and hamstring injury. Our search timeframe was from inception to January 2021. Key words (Table 1) were chosen in accordance with the aims of the research. Search terms were combined by Boolean logic (AND [between categories], OR [within categories]). Reference lists were also hand searched for any possible relevant studies.

### 2.2. Selection Criteria

Articles examining injury prevention programmes for the hamstrings were eligible for full-text review. An article was eligible for inclusion if it met all of the following inclusion criteria: (A) the article was an RCT, (B) included healthy athletes who participated within organized sports of either sex (C) included an intervention in comparison with a control or alternative intervention for the prevention of HSI, (D) interventions that aimed to increase strength of the hamstrings or associated structures. An article was excluded if it: (A) included athletes with existing, or under treatment for, lower-limb musculoskeletal injuries including HSIs, (B) focused on children <10 years as HSIs have been found to occur in youth team sport athletes at 9 years old and older [35], (C) includes non-athletic or participants who do not regularly participate in sports or (D) the article was not in English. All criteria were independently applied by the lead author (NJR) to the full text of the articles that passed the eligibility screening of titles and abstracts.

### 2.3. Quality Assessment

The methodological quality of individual studies was assessed using the Physiotherapy Evidence Database (PEDro) scale (http://www.pedro.fhs.usyd.edu.au, accessed on 28 September 2021). Results from individual study analysis of quality were used to identify common areas of methodological weaknesses across studies. The grading of studies was performed by NJR and SR independently, and any disagreements in scoring were discussed and concluded.

PEDro uses 11 criteria, and reviewed studies were awarded one point for each criterion that was clearly satisfied, for a potential maximum value of 10 points. Criteria included; (1) eligibility criteria reported; (2) random assignment; (3) concealed allocation; (4) groups similar at baseline regarding most important prognostic indicator; (5) blinding of participants; (6) blinding of therapists who administered the therapy; (7) blinding of assessors who measured key outcome; (8) measures of at least one key outcome were obtained from more than 85% of initial participants; (9) all participants received treatment or control condition as allocated; (10) results of between-group arithmetical comparisons are reported and (11) study provides point measures and measures of variability for at least one key outcome.

### 2.4. Statistical Analyses

Data, including counts and description of methods, were extracted manually from included studies. DerSimonian and Laird [36] random effects models were used for all analyses (meta-analyses and sub-group) to produce summary log odds ratios (LOR) and 95% confidence intervals (CIs). The weighted means difference percentage (WMD%) was calculated to represent the aggregated differences of each individual study weighted by their sample size, WMD% and the size of each plot are proportional to their sample size. Overall effects were identified and the test for overall effect identified via the Z statistic, the Z statistics were interpreted as trivial (<0.19), small (0.20–0.59), moderate (0.60–1.19), large (1.20–1.99), and very large (≥2.0) (Hopkins, 2002a). We used this model to be consistent with previously reported reviews on the same outcome [29,37].

Group analyses included LORs, 95% CIs and heterogeneity between groupings. To observe the effect of compliance upon HSI risk, selected articles were grouped via the novel thresholds of compliance: very high (>75.1%), moderate-high (50.1–75%), low-moderate (25.1–50%) and very low (<25%). A measure of intervention consistency was also identified, whereby the injury observation period was divided by the number of compliant sessions, i.e., number of prescribed sessions with respect to reported compliance, to attain an average number of weeks per session (<1 week/session, 1.01–3.00 weeks/session, >3.01 weeks/session). The effect of intervention modality was also observed within the group analyses.

Heterogeneity test statistics and their *p* values were used to assess consistency of reported LORs across studies and between interventions. I-squared statistic (I^2^) were used to describe the percentage of total variation across studies due to heterogeneity rather than chance alone with values >50% to indicate substantial heterogeneity. Significant heterogeneity was indicated with a *p* < 0.10. A higher *p* value was chosen to test for heterogeneity since these tests have low power particularly where there are few studies analyzed. The τ^2^ is reported to describe the pooled among-study variance of true effects, thereby reflecting the magnitude of heterogeneity.

Publication bias was evaluated by funnel plots and Egger’s test using the Rosenthal method [38]. A fail-safe number of effects was calculated to determine how many un-retrieved null effects would be needed to diminish the significance of the observed effects to *p* < 0.05. All analyses were conducted by one of the authors using Jamovi (Jamovi project (2018) Computer Software, Retrieved from https://www.jamovi.org, accessed on 11 May 2021).

## 3. Results

### 3.1. Search Results

**Figure 1:**
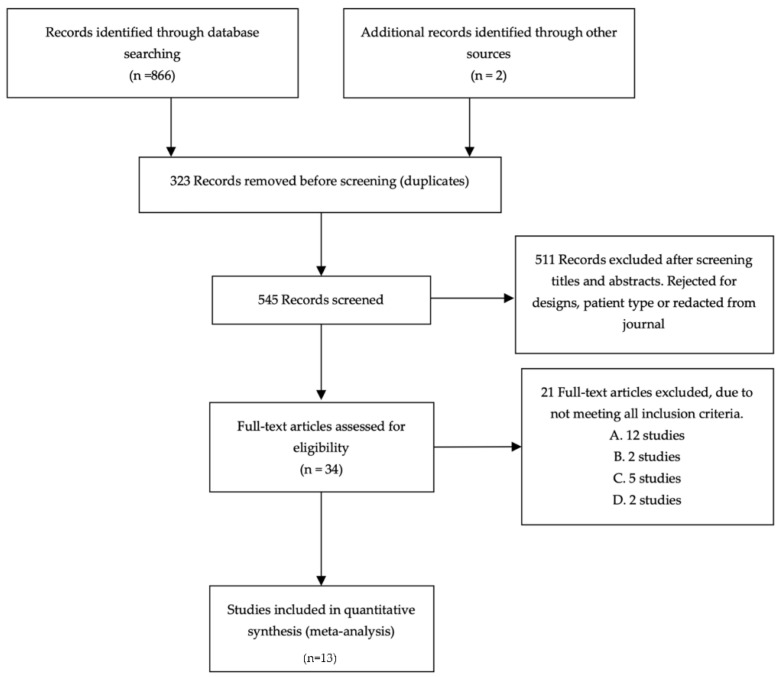
Preferred Reporting Items for Systematic Reviews and Meta-Analyses (PRISMA) flow diagram for study inclusion. Inclusion criteria A. Included athletes with existing, or under treatment for, lower-limb musculoskeletal injuries. B. Focused on children <10 years old. C. Includes non-athletic or participants who do not regularly participate in sports. D. Article was not in English.

Eight hundred and sixty-eight titles were identified through database and reference searches. Thirty-four full text articles were assessed for eligibility for inclusion, resulting in twenty-one studies being excluded based on study design and patient type, and a single study that was redacted by the journal. The process of study selection and the number of studies excluded at each stage, with reasons for exclusion is available in Figure 1.

### 3.2. Characteristics of the Included Studies

The number of athletes in the studies ranged from 30 [4] to 1892 [39]. A description of the included studies’ athlete populations, interventions, outcome measures, observation period and compliance are presented in Table 2.

### 3.3. Quality of Studies

The scores of the 11 criteria and total scores for each study are presented in Table 3.

### 3.4. Meta-Analysis Findings

The LOR, 95% CI and WMD% of hamstring injury following the implementation prevention protocol are presented in Table 2. The overall pooled estimate from the main effects analysis was −0.61 (95% CI −1.05 to −0.17). The test for overall effect favored the intervention treatments (Z = −2.70, *p* = 0.007). Heterogeneity was found between all studies (τ^2^ = 0.382 (standard error = 0.262), I^2^ = 67.66%, *p* = < 0.001).

**Figure 2:**
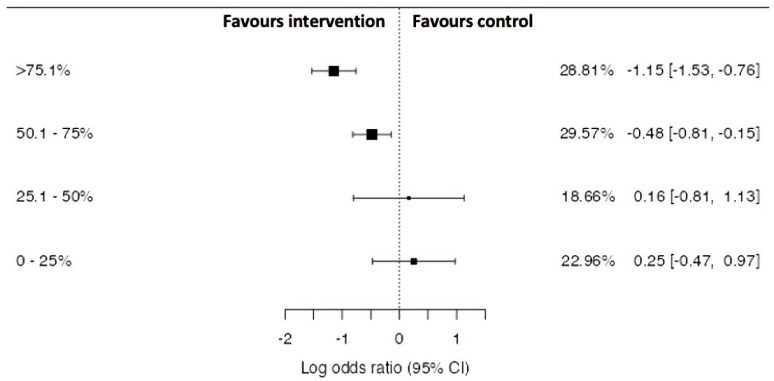
Comparison of intervention compliance rates on hamstring strain injury risk odds ratios based on grouped study estimates, 95% CI and WMD%.

The effect of intervention compliance on LOR, 95% CI’s and WMD% are demonstrated in Figure 2, with compliance was split into four sub-groups: very high compliance >75.1%, moderate-high compliance 50.1–75%, low-moderate compliance 25.1–50% and very low <25%. A non-significant, but large difference (*p* = 0.203, Z = −1.272) was demonstrated between all levels of compliance. With a meaningful trend of increased intervention, effectiveness can be observed with increased compliance, with both very high- and moderate-high-compliance interventions being more effective than both low-moderate- and very low-compliance.

**Figure 3:**
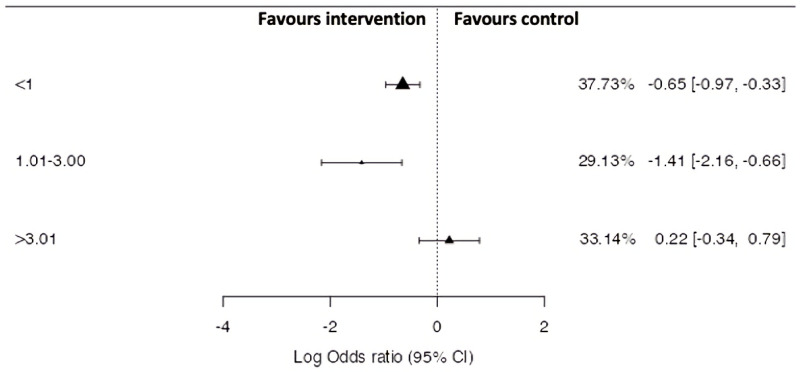
Comparison of average weeks per exposure on hamstring strain injury risk odds ratios based on grouped study estimates, 95% CI and WMD%.

Figure 3 illustrates the pooled effects between average intervention sessions per duration of study LOR, 95% CIs and WMD% on the probability of a HSI following the implementation of an intervention. A non-significant, but large difference (*p* = 0.137, Z = −1.488) was demonstrated between all levels of consistency. If the average weeks per session was greater than 3.01, then, the overall intervention effectiveness was negative (i.e., increased HSI occurrence). If there were less than 3 weeks between sessions there was a positive effect on HSI incidence, with the greatest positive effect on HSI occurring when sessions are performed every 1–2 weeks.

A significant difference was demonstrated between intervention modalities (*p* < 0.001, Z = −4.136). Interventions that prescribed eccentric hamstring strengthening on its own or as part of a series of exercises (FIFA 11+) were effective in reducing HSIs, in comparison to bounding intervention.

### 3.5. Bias Assessment

**Figure 4:**
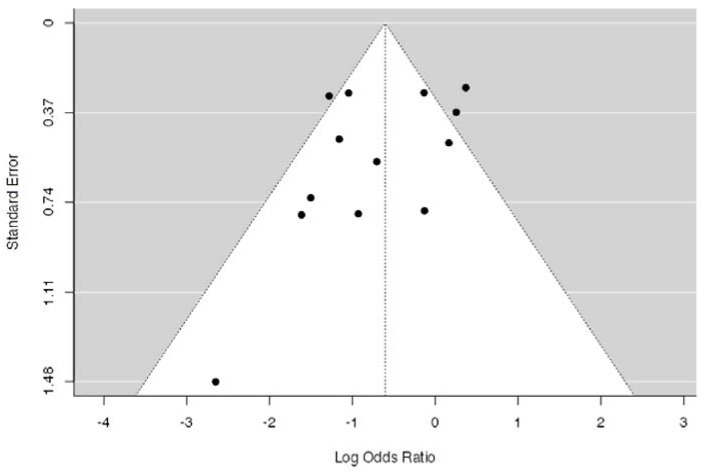
Funnel plot illustrating the publication bias results of the included studies.

The results of the Egger’s test suggest that the mean effect of HSI risk reduction interventions within the present meta-analysis are subject to publication bias (*p* < 0.001) with 93 “filed-away” studies needed to prove null effects. A funnel plot was used to visually assess symmetry and identify potential outliers (Figure 4).

## 4. Discussion

Within this systematic review and meta-analyses, we assessed the effect of compliance, consistency (average weeks between sessions) and intervention type on the strength and direction of pooled study estimates for the log odds ratios. Our search yielded 13 studies that met the inclusion criteria. Data from these sources demonstrated similar preventative effects towards HSI prevention as data reported by previous systematic reviews [18,29,32,37]. The results of the present review highlight that for HSI prevention measures to have a meaningful, positive effect upon HSI occurrence, a compliance of ≥50.1% should be achieved. Furthermore, with increased compliance (>75.1%) there is a 139% increase in intervention effectiveness. This provides novel and useful information surrounding the level of compliance that should be achieved by practitioners when implementing such interventions which could aid in understanding the effectiveness of an intervention. Furthermore, regular performance of the prevention measure yields greater positive effects in HSI prevention. Additionally, statistically significant preventative effects were observed for eccentric training, incorporating the NHE, and the implementation of the FIFA 11+ [18], whereas no significant preventative effect was observed for a bounding exercise programme. Using the PEDro assessment criteria, study quality varied between 5–7, many studies were limited by the ability blind participants, coaches or assessors, which is understandable in sports where it is obvious who the is performing the intervention or not especially when it is delivered within the same organization or club. Several studies were also limited by not achieving equivalent groups at baseline, with differences identified in physical performance [43], and between leagues [17,45,47].

Goode et al. [29] performed an intention-to-treat analysis to observe the effect of intervention compliance on hamstring injuries, it was demonstrated that following the removal of non-compliers from the analysis there was a substantial (65%) decrease in the risk of future HSI from eccentric training. A similar 65% reduction was observed in an observational intervention study following an eccentric NHE intervention [49]. More recently, Chebbi et al. [50] demonstrated that player compliance or attendance for NHE training of greater than 70% had a positive effect on reducing HSI incidence, with lower levels of compliance resulting in greater HSI rates within elite soccer players. The prescription of HSI prevention training, however, varied between seasons (20–53 sessions per season) [50], therefore, despite high frequency being an important factor, 70% attendance varied between seasons (14 vs. 37.1 sessions), so total exposure and consistency of exposure to the stimulus varied greatly between seasons. Although no detail on the exact prescription was provided including exercises, volumes, progressions and variations, which could have influenced the effectiveness of HSI prevention practices [50]. The results of the present review highlighted that inconsistent (>3 weeks between exposures) performance of prevention practices had a negative effect on HSI incidence, with a more positive outcome on HSI rates when practices are performed more regularly. This finding could be explained by the rapid loss in architectural and eccentric strength measures that have been identified as risk factors associated with the occurrence of HSIs, within as little as two weeks of training cessation [25,26,27,28]. This coincides with the effect of compliance, if a planned risk reducing practice cannot be regularly applied and consistently followed on a regular basis then the efficacy of that practice cannot be deemed successful. For HSI risk mitigation practices to be successful, they should therefore be easy to apply in sport, while also being able to consistently be followed without the risk of reducing compliance.

Common barriers to non-compliance in strength and conditioning and physiotherapy practices are commonly reported to include: DOMS [5], pain during exercise [51,52,53,54], confusion regarding correct exercise execution [54], and poor coach support [54]. Consistent with a previous review [29], DOMS was reported to be a main reason for non-compliance across several studies that were included within this review [7,39,41,42]. Gabbe et al. [40] identified that athletes may believe that DOMS increases their risk of future HSI, which would likely affect intervention compliance. Furthermore, the high volume of eccentric hamstring exercise prescribed within the interventions [4,17,39,41,42,46], could be a contributing factor in resultant DOMS and non-compliance [29]. More recently low volumes of the NHE have been shown to result in similar positive training adaptations which may contribute to the reduction in future HSI occurrence [26]. Furthermore, as the magnitude of the repeated bout effect is similar between high and low volumes of eccentric exercise [15]; the potential positive effects of low volume NHE training on HSI incidence could be hypothesised. Due to the similarity in repeated bout effect between eccentric exercise volumes, if eccentric volume is decreased there would be a decrease in muscle damage and thus resultant muscle soreness, but adaptation would likely still occur [15,55,56]. This indicates that intervention compliance maybe improved upon by the implementation of low volume eccentric hamstring exercises, as there would be a reduction in ensuing DOMS. A prospective cohort study by Seagrave et al. [7] identified that a critical minimum volume of the NHE being 3.5 repetitions per week may reduce the occurrence of HSIs within professional baseball players when compared to a control group, however, DOMS was still reported as major reason for non-compliance. One possible explanation for this non-compliance could be that the critical volume was the average number of completed repetitions across the season with no standardisation or structured programming, which may have resulted in several weeks of detraining followed by a single high-volume week resulting in a high degree of DOMS.

Athlete boredom and motivation were further identified as barriers to non-compliance to interventions [41]. One possible method of overcoming this maybe by providing direct supervision by trained professionals, who can offer encouragement and support [29]. Additionally, the use of novel devices that can provide real-time augmented feedback to the performance of tasks, such as the NHE, has the potential to increase athlete exertion (i.e., increased mean eccentric force [57]). An increase in athlete exertion is then likely to increase the adaptive response and in turn reduce the risk of HSI [25]. Several studies provided direct supervision of athletes, this included sports coaches or physical therapists, who were provided training by the investigators in how to perform the exercises prior to commencing the intervention, reporting moderate to very high levels of compliance (59.4–100.0%) [4,6,39,42,44,45,48]. Although the quality and reported compliance varied between the studies, the effect of regular and consistent feedback received from: sports coaches, strength and conditioning coaches, physical therapists, physicians, or peers, should not be underestimated in the role for a positive change. Although on-field supervision of the FIFA 11+ warm up intervention demonstrated only a minimal effect on performance of the intervention [58,59], there was a substantial difference in the volume of exercises performed [58,59]. Moreover, direct supervision could improve exercise quality, thus improving intervention effectiveness [29]. Additionally, improving athlete and coach education will aid in both debunking common beliefs (including that performing eccentric exercises may increase the risk of future HSIs [40]), and providing a greater understanding of the preventative value of their implementation [58,59] may assist in improving intervention compliance. Holm [60] identified that compliance may in fact be a problem of the practitioner, in this case instructing athletes to perform a practice in a paternalistic manner, where it might be more effective involve all parties (coach and athlete) equally in the decision-making process. Therefore, future research should be directed to the potential of low volume of eccentric strengthening exercises with an interest in compliance, as well as the potential of implementing other intervention protocols that may achieve greater athlete compliance e.g., sprint-based interventions [23].

Studies were also grouped by the average duration between prevention practice exposures, which accounted for the identified compliance to get a true picture of the overall performance. It was highlighted that two studies had very long average durations (>3.01 weeks) between exposures Engebretsen et al. [41] and Gabbe et al. [40] implemented extremely high-volume protocols, Mjolsnes protocol [5] and 12 sets of six [40], respectively. These higher volume interventions can result in excessive fatigue and DOMS, with both factors having a negative impact on overall compliance, and with observed compliance rates of 21.1% and 47.0% [40,41]. The consistency of intervention application can also be questioned; Gabbe and colleagues’ [40] protocol incorporated five training sessions across a 12-week period, whereby multiple weeks could pass prior to the subsequent dose with a long observation period following the final session. This becomes an issue as the structural and force producing capabilities of the hamstrings can rapidly return to baseline in as little of two weeks [25,26,27], and therefore potentially lose their preventative adaptations. Additionally, long durations between exposure to eccentric muscle damage can limit the effectiveness of the repeated bout effect, which aids in reducing DOMs for subsequent sessions [15,61,62]. Studies that were identified as having a moderate training consistency (1.01–3.00 weeks between sessions) demonstrated a positive effect on HSI incidence [4,42]; however, due to the longer durations between session there could have been detrimental effects on the architectural and force producing capabilities of the hamstrings, as detraining has been observed in as little as two-weeks [25,26,27]. Although these studies performed a training phase lasting between 10–13 weeks, which was then followed up with an observation phase that lasted between 36–42 weeks, the average duration between sessions was negatively affected as all exposures were condensed to a single initial training period. There are, however, several explanations that might explain why these interventions remained highly effective, despite poor consistency. Firstly, these studies had very high levels of compliance (91–100%); additionally, they were also performed during “breaks” within the normal season, which may have influenced athlete motivation and subsequent adaptation.

Unsurprisingly, the intervention types that were most effective at decreasing the occurrence of future HSI included eccentric exercise and FIFA 11/FIFA11+ warmups. While bounding, provided a minimal decrease in the risk of future HSI occurrence, as although the observed LORs are less than zero (−0.14), the 95% CI includes zero. Within a recent review and meta-analyses, eccentric hamstring training (i.e., NHE), has been found to decrease the risk of injury by up to 50% [30], although this has been identified as being potentially misleading [32]. This is potentially as a result of the positive adaptations that have been shown to occur following their implementation, including increased bicep femoris long head fascicle length and increased force production across muscle actions, joint angles and movement velocities [5,26,56,63,64]. No research to date has demonstrated what adaptations may occur from the implementation of the FIFA 11 and FIFA 11+ that may aid in hamstring injury prevention. Nevertheless, the warmup interventions still offer a positive effect on the risk of future HSI occurrence, making it an effective, practical and time efficient practice in sport. The bounding intervention, with the inclusion of dynamic lunges and bounding variations over incremental distances, implemented by van de Hoef et al. [65], may not have elicited a desired preventative effect as hypothesised, as the magnitude of hamstring loading may have not been a sufficient stimulus for an adaptive response to occur, although no measure of strength or muscle architecture was taken [65].

The current review is not without methodological limitations. Firstly, only one author was involved in the study selection process which could have resulted in individual bias or error within the study selection process; however, similar search strategies that have been reported within previous systematic reviews as recent as May 2021 were used [18,29,30,32]. Using the previously reported search strategies, a similar volume of records was discovered, which eventually resulted in all articles which have been reported previously, having been discovered along with more recent literature. Within the current review, effects were pooled into subgroups by intervention compliance, consistency and modality, and this is without the removal of possible study outliers identified by funnel plot [41,47], potentially impacting on the determined effects. However, the removal of study outliers would be contraindicated as both studies still offer an insight into HSI risk reduction strategies within sport and the possibility of null effects. Furthermore, the funnel-shaped plot (Figure 4), illustrating the observed effects vs. the standard error can be disrupted by the heterogeneity of the studies, thereby increasing the likelihood of false-negative and false-positive decisions about publication bias [66]. Intention-to-treat analysis has been described as the preferred method of determining effectiveness of interventions in RCT [29], yet can be subject to null-bias where substantial non-compliance is reported [29]. However, as intention-to-treat analysis has been performed previously within a similar review [29], and the aim of this review was to observe the effect of total intervention compliance providing a novel scale of very high (>75.1%), moderate-high (50.1–75%), low-moderate (25.1–50%) and very low (<25%) compliance on the observed effect, it was deemed unnecessary. The data extraction from the literature identified within the defined search strategy was limited due to a lack of detailed reporting of intervention compliance; hence, a unit of average weeks/session was identified despite being limited by some study designs using short intervention periods with large observation periods. Future research should look to provide a more detailed explanation of the distribution of training across the study timelines, and furthermore, individual training compliance data could allow for more accurate reporting of the effectiveness of an intervention with regards to compliance.

## 5. Conclusions

In conclusion the results of the present systematic review and meta-analysis demonstrate that the effectiveness of interventions is related to training compliance, with an increase in compliance resulting in greater effectiveness. Compliance of >50.1% demonstrated a positive effect on the occurrence of future HSI. Crucially, further increases in compliance (>75.1%), resulted in an 139% increase in preventative effect, highlighting the need for practitioners to design and implement interventions whereby a compliance of >75.1% is achievable. A similar finding was observed for consistency of training application, with an average of <3 weeks per exposure having positive beneficial effects on HSI incidence. Therefore, training interventions that can achieve both high levels of compliance, and can be consistently performed, should be the main objective of any future practice or intervention. As per previous systematic reviews and meta-analyses, eccentric resistance training and the FIFA 11+ are effective at decreasing HSI incidence, although it has been highlighted that the evidence for the NHE is inconclusive and can only be conditionally recommended [32]. A bounding intervention offered limited positive protection to the occurrence of future hamstring injury. However, only a single intervention has utilized this methodology and therefore requires further investigation. Future studies should also investigate other alternative methods that are currently being employed in practice but currently do not have supporting research, such as sprinting and isometric static and dynamic exercises, which may lead to similar positive adaptations to the modifiable risk factors, while promoting athlete buy-in by reducing potential DOMS and increasing competition, in order to achieve desirable levels of compliance (>75.1%). With regards to eccentric resistance exercise (e.g., NHE), volume prescriptions in research and practice (including the FIFA11 and 11+) continually appear to be higher than what might be tolerable for the majority of athletes. Hence it is recommended that prescriptions should be of low volumes (1–2 sets × 2–4 repetitions, 1–2 per week, with a progressive intensity (i.e., the addition of load) [26,28,67]), which are sufficient enough to have a positive effect on hamstring architecture, strength and HSI incidence, while engaging the repeated bout effect to minimize reoccurrence of DOMS in subsequent exposures [15], without being initially overly demanding or damaging (i.e., minimal DOMS), which could offset the lower observed compliance in some of these investigations.

## Figures and Tables

**Table 1:** Summary of keyword grouping employed during database searches.

Injury	Prevention	Training	Study
Hamstring strain injury	Injury prevention	Resistance training	Randomised control trial (RCT)
Hamstring injury	Hamstring injury prevention	Strength training	Sport
Posterior thigh injury	Primary prevention	Eccentric	Team sport
Lower extremity strain	Injury prevention programmes	Eccentric training	Soccer
Lower limb injury	Injury risk reduction	Nordic hamstring exercise	
	Compliance	Nordics	
		Warm up	
		FIFA 11	
		FIFA 11+	
		Plyometrics	
		Sprinting	

**Table 2:** Summary of athletes, interventions, comparators, percentage compliance and injuries of included studies.

Reference	Population	Intervention Description	Sessions Planned	Compliant Sessions Completed	Weeks per Session	Observation Period	n Intervention	n Control	Number of Injuries Intervention	Number of Injuries Control	Compliance (%)	Log Odds Ratio (95% CI)	Weighted Mean Difference (%)
Gabbe et al. [40]	Male Australian amateur soccer players	NHE training intervention (12 × 6)	5	2.4	15.3	1 season	114	106	10	8	47.0%	0.16(−0.81 to 1.13)	8.10%
Engebretsen et al. [41]	Male Norwegian soccer players	10-week NHE training intervention from Mjølsnes et al. (2004)	27	5.7	6.3	1 season	85	76	23	17	21.1%	0.25 (−0.47 to 0.97)	9.81%
Askling, Karlsson and Thorstensson [4]	Male Swedish soccer players	10-week eccentric training intervention (“Yo-Yo” ergometer)	16	16.0	2.9	46 weeks	15	15	3	10	100.0%	−1.50 (−2.92 to–0.09)	5.62%
Van der Horst et al. [42]	Male Dutch amateur soccer players	13-week NHE training intervention from Mjølsnes et al. (2004)	25	22.8	2.3	12 months	292	287	6	18	91.0%	−1.16 (−2.10 to −0.22)	8.30%
Sebelien et al. [43]	Male Semi-professional soccer players	Progressive NHE training (2 × 5, 1/week–3 × 8–10, 2/week)	102	23.2	1.6	1 season	59	60	0	6	22.7%	−2.65 (−5.55 to 0.25)	1.97%
Petersen et al. [6]	Male Danish soccer players	10-week NHE training intervention from Mjølsnes et al. (2004)	67	61.0	0.8	12 months	461	481	15	52	91.0%	−1.28 (−1.87 to −0.69)	10.47%
Silvers-Granelli et al. [17]	Male NCAA collegiate athletes	FIFA 11+ three times per week.	60	28.2	0.7	5 months (August–December)	675	850	16	55	47.0%	−1.05 (−1.61 to −0.48)	10.90%
van de Hoef et al. [44]	Male Dutch amateur soccer players	Bounding exercise programme.	74	52.5	0.7	1 season (9 months)	229	171	31	26	71.0%	0.14 (−0.70 to 0.43)	10.92%
del ama-espinosa et al. [45]	Female Elite European soccer players	NHE training intervention, 1 × 5 performed once per week for 42 weeks.	42	33.6	0.7	1 season	22	21	3	6	80.0%	−0.93 (−2.47 to 0.61)	5.07%
Van Beijsterveldt et al. [46]	Male Dutch amateur soccer players	FIFA 11 warm up twice per week.	74	54.0	0.7	1 season (9 months)	223	233	18.4% [38]	13.4% [29]	73.0%	0.37 (−0.15 to 0.89)	11.20%
Saleh et al. [47]	Male Australian amateur soccer players	Additional FIFA 11+ performed post-exercise two-three times per week.	48	39.8	0.6	6 months	144	136	2	9	83.0%	−1.62 (−3.17 to −0.06)	5.03%
Hasebe et al. [48]	High school male soccer players	Progressive continual NHE training (5 × 2, 1/week–8–10 × 3, 2/week)	53	46.6	0.6	27 weeks	156	103	4	3	88.0%	−0.13 (−1.65 to 1.39)	5.17%
Soligard et al. [39]	Youth female soccer players	FIFA 11+ warm up, 2/week.	148	87.9	0.4	1 season	1055	837	5	8	59.4%	−0.71 (−1.83 to 0.41)	7.16%

NHE = Nordic hamstring exercise. NCAA = National collegiate athletic association.

**Table 3:** The Physiotherapy Evidence Database (PEDro) quality assessment of individual studies.

Reference	1 *	2	3	4	5	6	7	8	9	10	11	Total Score
Askling, Karlsson and Thorstensson [4]	-	X	-	X	-	-	X	X	X	X	X	7
Engebretsen et al. [41]	X	X	-	X	-	-	-	X	X	X	X	6
Petersen et al. [6]	X	X	X	X	-	-	-	X	X	X	X	7
Van der Horst et al. [42]	X	X	X	X	-	-	-	X	X	X	X	7
Gabbe et al. [40]	X	X	-	X	-	-	-	-	-	X	X	4
Sebelien et al. [43]	X	X	X	-	-	-	-	X	X	X	X	6
del ama-espinosa et al. [45]	X	-	X	-	-	-	X	X	-	X	X	6
Saleh et al. [47]	X	X	X	-	X	-	-	X	X	-	X	6
Silvers-Granelli et al. [17]	X	X	X	-	-	-	X	X	-	X	X	6
Van Beijsterveldt et al. [46]	-	X	X	X	-	-	-	X	-	-	X	5
Soligard et al. [39]	X	X	X	-	-	-	X	-	-	X	X	5
van de Hoef et al. [44]	X	X	-	X	-	-	-	-	X	X	X	5
Hasebe et al. [48]	X	X	X	X	-	-	-	X	X	X	X	7

1. Eligibility criteria were specified. * Does not contribute to total score. 2. Subjects were randomly allocated to groups. 3. Allocation was concealed. 4. Groups were similar at baseline regarding most important prognostic indicators. 5. Blinding of all participants. 6. Blinding of coaches who administered the intervention. 7. Blinding of all assessors who measured at least one key therapy. 8. Measures of at least one key outcome obtained from more than 85% of the participants. 9. All subjects for whom outcome measures were available received the treatment or control condition as allocated. 10. Results of between-group statistical comparisons are reported for at least one key outcome. 11. Study provides both point measures of variability for at least one key outcome. X, met criteria; -, criteria not met.
